# Temporal trends in outpatient right colectomy: a contemporary multistate study

**DOI:** 10.1007/s00464-025-11888-x

**Published:** 2025-07-14

**Authors:** Drew W. Goldberg, Shane Williams, James Sharpe, Joshua Bleier, Nicole Saur, Rachel R. Kelz

**Affiliations:** 1 Department of Surgery, Center for Surgery and Health Economics, University of Pennsylvania, 3400 Spruce Street, 4 Silverstein, Philadelphia, PA 19104 USA; 2 Leonard Davis Institute of Health Economics, University of Pennsylvania, Philadelphia, PA USA; 3 Department of Surgery, Perelman School of Medicine, University of Pennsylvania, Philadelphia, PA USA; 4 Division of Colorectal Surgery, University of Pennsylvania, Philadelphia, PA USA

**Keywords:** Colectomy, Outpatient surgery, Health services, Cost

## Abstract

**Introduction:**

Outpatient colectomy has been shown to be safe and effective in small, single or multicenter institutional series. Early discharge following colorectal surgery has been made possible, in part, by standardization of perioperative care and postoperative monitoring. We examined modern national trends in the use of outpatient right colectomy (ORC) to inform future practice patterns.

**Methods:**

We performed a retrospective cohort study of adult patients undergoing elective ORC using the Healthcare Cost and Utilization Project State Inpatient Databases and State Ambulatory Surgery and Services Databases from 2016 to 2021. Data were linked to the American Hospital Association survey to calculate cost in 2021 US dollars. Outpatient surgery was defined as a length of stay ≤ 1 day. The primary outcomes were the annual frequency of ORC and annual proportion of ORC per total colectomies. The Cochran-Armitage test of trend was used to compare annual frequency and proportion of ORC. Regression models were used to assess total admission cost and 30-day readmission by year. Spearman’s correlation assessed annual trends.

**Results:**

Of 49,416 right colectomies meeting the study criteria, 1933 (3.9%) underwent ORC. The ORC median [IQI] patient age was 63 [54, 70] years and 85.8% underwent a minimally invasive approach. There was a significant increase in the annual frequency (*p* = 0.03) and proportion (*p* < 0.001) of ORC over time. There were no significant differences in 30-day readmission rates over time (*p* = 0.44). A significant increase in risk-adjusted yearly total cost for ORC was identified, ranging from $9,447 in 2016 to $14,544 in 2021 (Spearman’s correlation test rho = 1, *p* = 0.003).

**Conclusion:**

In a contemporary geographically representative cohort, the practice of ORC increased over five years without differences in readmission rates. ORC cost increases potentially indicate an increased resource requirement to provide acute postoperative care in the ambulatory setting.

**Supplementary Information:**

The online version contains supplementary material available at 10.1007/s00464-025-11888-x.

Enhanced perioperative care and postoperative monitoring have improved the ability to perform surgery in the ambulatory space [1]. Beginning with the implementation of Enhanced Recovery After Surgery protocols in colorectal surgery in the early 2000s [2], and more recently with improved techniques in minimally invasive surgery (MIS) and telehealth monitoring, surgical patients’ length of stay have seen dramatic decreases in the last two decades [3, 4]. Moreover, external factors such as value-based reimbursement schemes [5] and strains on healthcare resources, especially during the COVID-19 pandemic, have catalyzed the transition of surgery into the outpatient setting in recent years [6–8].

Despite being first described as safe over 15 years ago [9], outpatient colectomy—conventionally defined by discharge within 24 h following surgery—continues to be utilized in a limited fashion [10]. Nonetheless, multiple groups have demonstrated that with the appropriate infrastructure, patient selection, and support, this approach can be feasible and safe for patients [1, 11–14]. In addition to improved outcomes, there are significant potential cost savings from such initiatives [10, 15]. However, to date, published series are limited in number of enrolled patients, number of sites, and analysis of extended time periods.

We therefore aimed to assess recent trends in the utilization, cost and outcomes of outpatient right colectomy. To do this, we used a multistate database to track temporal changes in the use of outpatient colectomy over six years. We hypothesized that utilization and cost would increase, while readmissions would decrease over the study period.

## Materials and methods

### Data and study population

We performed a retrospective cohort study of adult patients (≥ 18 years old) undergoing elective right colectomy (open or MIS), 2016 to 2021, using the Healthcare Cost and Utilization Project (HCUP) State Inpatient Databases (SID) and State Ambulatory Surgery and Services Databases (SASD) from Florida, Iowa, Maryland, Nebraska, Utah, Vermont and Wisconsin. These states were selected because they permit the tracking of patients over multiple admissions. SID contains inpatient discharge information from community hospitals within a given state, while SASD contains data from hospital and non-hospital affiliated facilities. Exclusion criteria included patients treated in the non-elective setting and those without complete readmission data (HCUP “visitLink”). For patients with multiple index admissions during the study period, the first admission was included with subsequent hospitalizations included only if they met criteria for readmission. Patients experiencing death prior to discharge were excluded given we could not determine their true length of stay (LOS). Primary diagnoses associated with a procedural episode were extracted using International Classification of Disease, version 10 (ICD-10). HCUP data were linked to the American Hospital Association (AHA) survey to calculate inflation adjusted cost relative to 2021 US dollars [16]. The total cost of each admission was calculated by multiplying the total charge for each admission by each hospital’s cost-to-charge ratio and then adjusting for geographic variation through the wage index. Furthermore, all costs were converted to 2021 dollars using the US Consumer Price Index for medical care.

### Exposure

Right colectomy was used as the primary exposure of interest, as this colectomy type is associated with fewer complications such as anastomotic leak or surgical site infection compared to left sided resections, therefore providing the highest potential yield of cases in the outpatient setting [17]. Right colectomy (open or minimally invasive) was defined using ICD-10 procedure codes and Current Procedural Terminology codes (Supplemental Table 1). Patient were considered to have undergone outpatient right colectomy (ORC) if they had a LOS ≤ 1 day. Patients with LOS > 1 day were considered to have undergone inpatient surgery.

**Table 1:** Right colectomy trends by year, 2016 to 2021

	All years	2016	2017	2018	2019	2020	2021	*p*-value
Outpatient colectomy, *N*	1933	245	247	321	368	370	382	
Open, *N* (%)	278 (14.2)	21 (8.6)	36 (14.6)	42 (13.1)	49 (13.3)	62 (16.8)	68 (17.8)	0.03**
Minimally invasive, *N* (%)	1658 (85.8)	224 (91.4)	212 (85.8)	279 (86.9)	320 (86.9)	309 (83.5)	314 (82.2)	0.03**
Inpatient colectomy*, *N*	47,483	8402	8570	8124	7957	6923	7507	
Total colectomy, *N*	49,416	8647	8817	8445	8325	7293	7889	
Proportion outpatient to total colectomies, %	0.04	0.03	0.03	0.03	0.04	0.05	0.05	< 0.01***

^*^Length of stay > 1 day

^**^Chi-squared test

^***^Cochrane-Armitage test

### Covariates

Patient-level covariates collected included age, race, ethnicity, sex, 38 Elixhauser comorbidities [18], insurance status, median household income quartile (based on patient zip code), patient distance to hospital (the geodetic distance, in miles, between the centroids of the patient zip code and the hospital zip code), and surgical technique (open versus MIS). Race was categorized as White, Black, or other/missing. Ethnicity was categorized as Hispanic, non- Hispanic, or missing ethnicity. Sex was categorized as female or male. Insurance status was categorized as Medicare, Medicaid, private insurance, or other/missing. Missing data from continuous covariates were excluded from statistical analyses, while missing data from categorical covariates were included in a separate category.

### Outcomes

The primary outcome was the annual proportion of right colectomies performed in the outpatient setting over all right colectomies performed in that year. The frequency of yearly ORC by surgical approach was also tabulated. Secondary outcomes included annual inflation-adjusted index cost, LOS, and 30-day readmission rate.

### Analysis

The baseline characteristics were compared among each annual cohort of ORC patients. Summary statistics are presented as the median with corresponding interquartile interval (IQI). Continuous variables were compared using the Kruskal–Wallis test, while categorical variables were compared with Fisher’s exact or Chi-squared tests. We compared the rate of the categorical variables and the medians of continuous variables across each year. The yearly rate of outpatient colectomy performed was analyzed using the Cochran-Armitage test of trend and plotted to visualize the temporal trends. Unadjusted analysis also used the univariate methods to compare secondary outcomes per year. Secondary outcomes were further analyzed using generalized logistic regression (binary outcomes) or a generalized linear regression (continuous outcomes) to associate procedural year with secondary outcomes with adjustment for relevant patient characteristics. Post-estimation routines were used on these models to find the predictive margins of each outcome by the years in the study period. The Spearman's correlation test was applied to assess a positive or negative correlation between the predictive margins for the outcomes and the year. Models included admission year, state, race, sex, insurance class, income quartile, surgical technique, and Elixhauser comorbidities (Acquired Immune Deficiency Syndrome, alcohol abuse, iron deficiency anemia, arthropathy, chronic blood loss anemia, lymphoma, leukemia, metastatic cancer, solid tumor in-situ, solid tumor with malignancy, cerebrovascular disease, congestive heart failure, coagulopathy, dementia, depression, diabetes, drug abuse, hypertension, liver disease, chronic lung disease, movement disorder, other neurologic disorder, seizures, obesity, paralysis, peripheral vascular disease, psychoses, pulmonary circulation disease, renal failure, hypothyroidism, other thyroid disorder, peptic ulcer disease, valvular disease, and weight loss).

All analyses and graphics were generated in SAS (version 9.4, SAS Institute, Cary, NC), R (version 4.4.1, R Core Team, Vienna, Austria, 2017) or STATA (version 17.0, 2021, College Stations, TX, USA, 2021). The two-sided statistical level of significance was set at 0.05. The University of Pennsylvania Institutional Review Board approval (protocol ID: 858113) was obtained prior to the start of this study. The STROBE guidelines were followed for reporting in this study [19].

## Results

### Patient characteristics

**Fig. 1:**
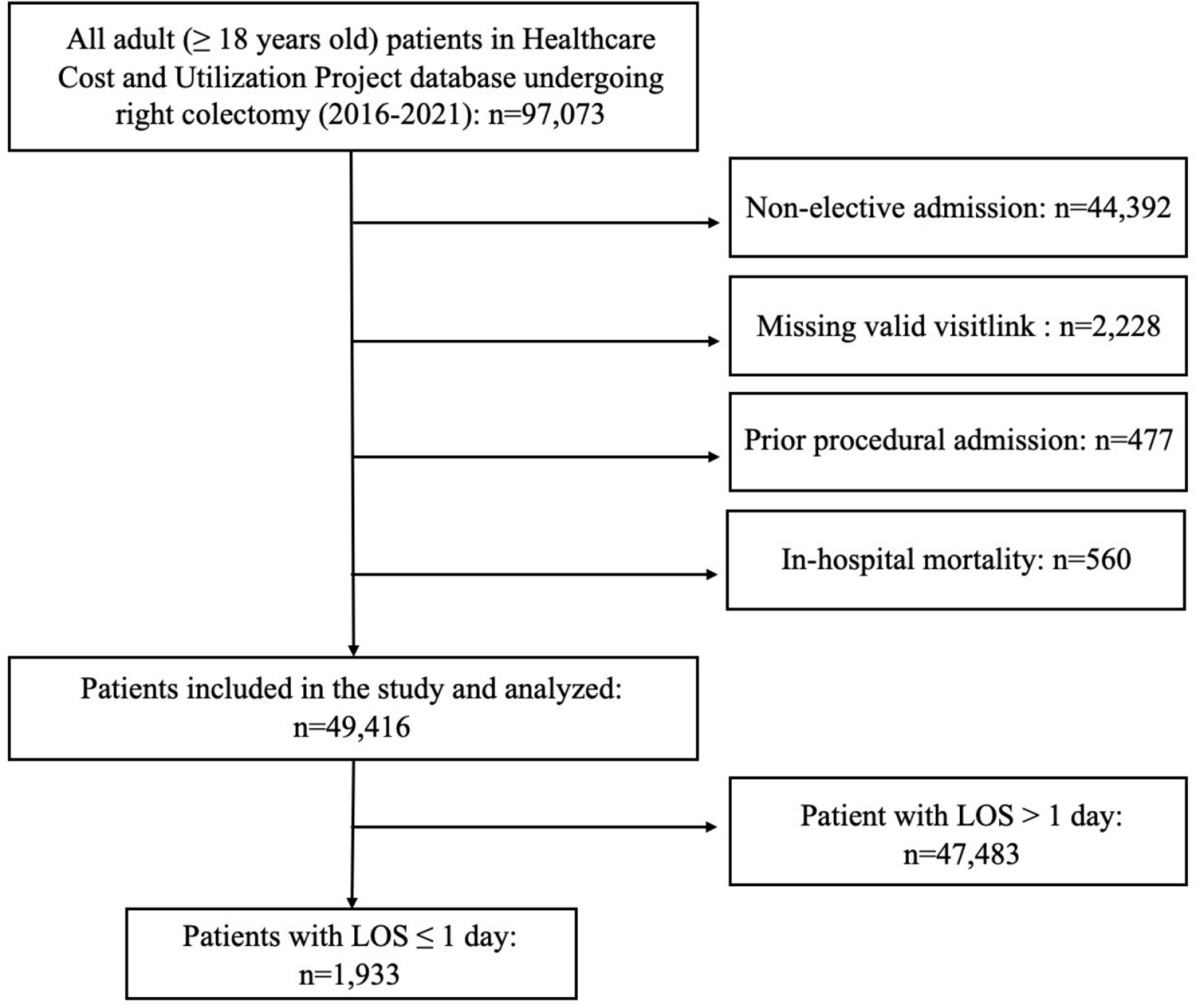
Patient inclusion diagram

A total of 49,416 patient were identified to have undergone a right colectomy during the study period. There were 1933 (3.9%) who had an outpatient operation with a LOS ≤ 1 day (Fig. 1). There were no significant differences between the proportion of patients with LOS 0 day versus LOS 1 day (*p* = 0.06) (Supplemental Table 2). Among ORC patients, 1,658 (85.8%) were performed via MIS approach and 278 (14.2%) were performed in an open fashion (Table 1).

**Table 2:** Patient demographic and comorbidity data, by year

	All years	2016	2017	2018	2019	2020	2021
Age, median years [IQI]	63 [54, 70]	63 [55, 70]	64 [54, 70]	63 [54, 70]	63 [53, 71]	63 [55, 71]	63 [55, 70]
Race, *N* (%)							
White	1492 (77.2)	191 (77.9)	186 (75.3)	245 (76.3)	287 (77.9)	290 (78.4)	293 (76.7)
Black	124 (6.4)	15 (6.1)	13 (5.3)	20 (6.2)	29 (7.9)	21 (5.7)	26 (6.8)
Other/missing	317 (16.4)	39 (15.9)	48 (19.4)	56 (17.5)	52 (14.1)	59 (15.9)	63 (16.5)
Ethnicity, *N* (%)							
Hispanic	123 (6.4)	16 (6.5)	18 (7.3)	22 (6.9)	17 (4.6)	22 (5.9)	28 (7.3)
Non-Hispanic	1630 (84.3)	206 (84.1)	198 (80.1)	268 (83.5)	322 (87.2)	313 (84.6)	324 (84.8)
Missing	180 (9.3)	23 (9.4)	31 (12.6)	31 (9.7)	30 (8.2)	35 (9.5)	30 (7.85)
Sex, female (%)	1034 (53.5)	134 (54.7)	132 (53.4)	177 (55.1)	188 (51.1)	209 (56.5)	194 (50.8)
Insurance							
Medicare	831 (42.9)	95 (38.8)	106 (42.9)	142 (44.2)	161 (43.8)	156 (42.2)	171 (44.8)
Medicaid	68 (3.5)	*	*	*	*	*	*
Private	987 (51.1)	132 (53.9)	126 (51)	158 (49.2)	193 (52.5)	192 (51.6)	187 (48.9)
Other/missing	48 (2.5)	*	*	*	*	*	*
Median household income quartile, *N* (%)							
1st	383 (19.2)	42 (17.4)	44 (17.9)	58 (18.2)	74 (20.1)	84 (22.7)	81 (21.5)
2nd	426 (22.2)	43 (17.8)	54 (22)	81 (25.4)	71 (19.3)	89 (24.1)	88 (23.3)
3rd	550 (28.6)	82 (33.9)	71 (28.9)	79 (24.8)	120 (32.6)	96 (25.9)	102 (27.1)
4th	562 (29.3)	75 (30.9)	76 (31)	101 (31.7)	103 (27.9)	101 (27.3)	106 (28.1)
Patient distance to hospital, median miles [IQI]	7.6 [3.8, 15.9]	6.5 [3.4, 14.7]	7.4 [4.1, 15.3]	8 [4.3, 16.2]	7.6 [3.8, 14.9]	8.8 [3.6, 15]	8.1 [3.2, 38.3]
Sum of Elixhauser comorbidities, median *N* [IQI]	1 [0, 2]	1 [0, 2]	1 [0, 2]	1 [0, 2]	1 [0, 2]	1 [0, 2]	1 [0, 2]
Elixhauser count, *N* (%)							
Iron deficiency anemia	112 (5.8)	*	*	20 (6.2)	16 (4.4)	28 (7.6)	28 (7.3)
Diabetes, uncomplicated	234 (12.11)	26 (10.6)	30 (12.2)	39 (12.2)	47 (12.8)	43 (11.6)	49 (12.8)
Hypertension, uncomplicated	766 (39.63)	96 (39.2)	92 (37.3)	137 (42.7)	139 (37.8)	146 (39.5)	156 (40.8)
Chronic lung disease	234 (12.11)	25 (10.2)	37 (15)	42 (13.1)	49 (13.3)	38 (10.3)	43 (11.3)
Obesity	303 (15.68)	31 (12.7)	34 (13.8)	57 (17.8)	56 (15.2)	55 (14.0)	70 (18.3)

All *p*-values > 0.05

*IQI* interquartile interval

^*^Data field contains observations < 11 and is censored in compliance with the HCUP data use agreement

The median [IQI] age of all patients who underwent ORC was 63 [54, 70] years, with 77.2% White (*n* = 1508), 6.3% Hispanic (*n* = 124), and 53.3% female (*n* = 1041). Among ORC patients, 43.2% (*n* = 845) had Medicare and 50.8% (*n* = 993) had private insurance (Table 2). Patients had a median [IQI] Elixhauser comorbidity count of 1 [0, 2]; approximately 32% of patients had no comorbidities. The most frequent diagnoses included benign neoplasm of the cecum (*n* = 418, 21.6%), malignant neoplasm of the ascending colon (*n* = 148, 7.7%), benign neoplasm of the appendix (*n* = 142, 7.4%), colonic polyp (*n* = 113, 5.9%), and benign neoplasm of the ascending colon (*n* = 110, 5.7%) (Table 3).

**Table 3:** Most frequent diagnoses associated with encounter for outpatient right colectomy

Diagnosis	Location	Percent
Benign neoplasm		
	Appendix	7.4
	Cecum	21.6
	Ascending	5.7
	Transverse colon	2.8
	Not specified	7.2
Malignant neoplasm		
	Appendix	2.8
	Cecum	5
	Ascending	7.7
	Hepatic flexure	1.1
	Transverse colon	2.3
	Not specified	1.4
Diverticulitis		
	Not specified	2.3
Other (e.g., appendicitis)		
	Appendix	6.1
	Not specified	5.5
Total		78.9

There were no significant differences in baseline demographics and comorbidities across the ORC groups by year (Table 2, Supplemental Table 3).

### Trends

**Fig. 2:**
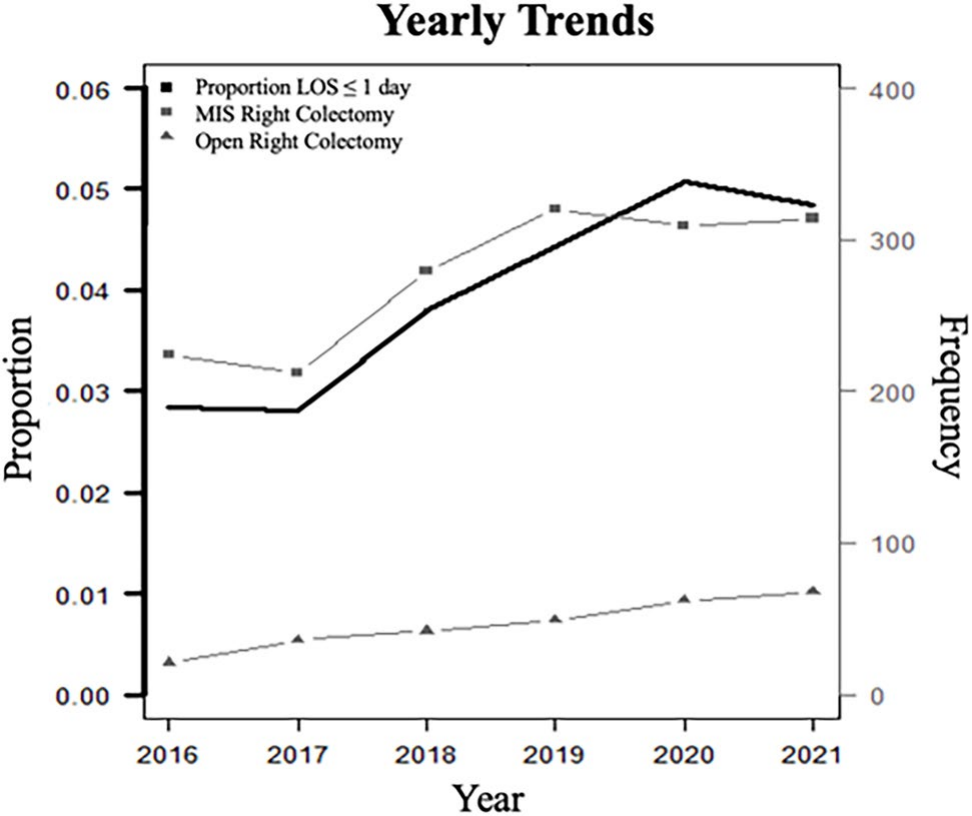
Yearly trends in outpatient right colectomy, by frequency and proportion. Cochran-Armitage test of trend in proportion of right colectomy (frequency of outpatient: frequency of outpatient plus inpatient) (p < 0.001). *LOS* length of stay; *MIS* minimally invasive surgery

There was a continuous increase in the frequency of annual ORC performed over the study period for both MIS (*p* = 0.03) and open (*p* = 0.03) colectomies. There was also a significant year-over-year increase in the proportion of colectomies performed in the outpatient setting (*p* < 0.001) (Table 1). Figure 2 demonstrates that the rate of outpatient MIS colectomies outpaced that of open colectomies.

### Unadjusted outcomes

Among ORC patients, the median [IQI] length of stay was 1 [0, 1] days with a median index admission cost of $9,521 [$7,106, $12,730] (Table 4). Median index admission cost [IQI] was significantly different across the study years; 2016: $8,539 [$6,896, $10,822]; 2017: $8,943 [$6,788, $11,384]; 2018: $9,121 [$7,040, $11,943]; 2019: $10,027 [$7,248, $12,868]; 2020: $10,855 [$7,906, $14,675]; 2021: $11,293 [$8,166, $14,626]; p < 0.001. Across the entire study period, the 30-day readmission rate was 12.9%. Across the years, the 30-day readmission rates were similar (2016: 11.4%; 2017: 13.8%; 2018: 13.4%; 2019: 16%; 2020: 12.4%; 2021:10.5%; *p* = 0.31).

**Table 4:** Unadjusted outcomes

	All years	2016	2017	2018	2019	2020	2021	*p*-value
Index encounter costs, median 2021 USD [IQI]	9521 [7106, 12730]	8,539 [6,896, 10,822]	8,943 [6,788, 11,384]	9,121 [7,040, 11,943]	10,027 [7,248, 12,868]	10,855 [7,906, 14,675]	11,293 [8,166, 14,626]	< 0.001
30-day readmission, *N* (%)	250 (12.9)	28 (11.4)	34 (13.8)	43 (13.4)	59 (16)	46 (12.4)	40 (10.5)	0.31

*IQI* interquartile interval, *LOS* length of stay, *SD* standard deviation, *USD* United States dollars

### Adjusted outcomes

**Fig. 3:**
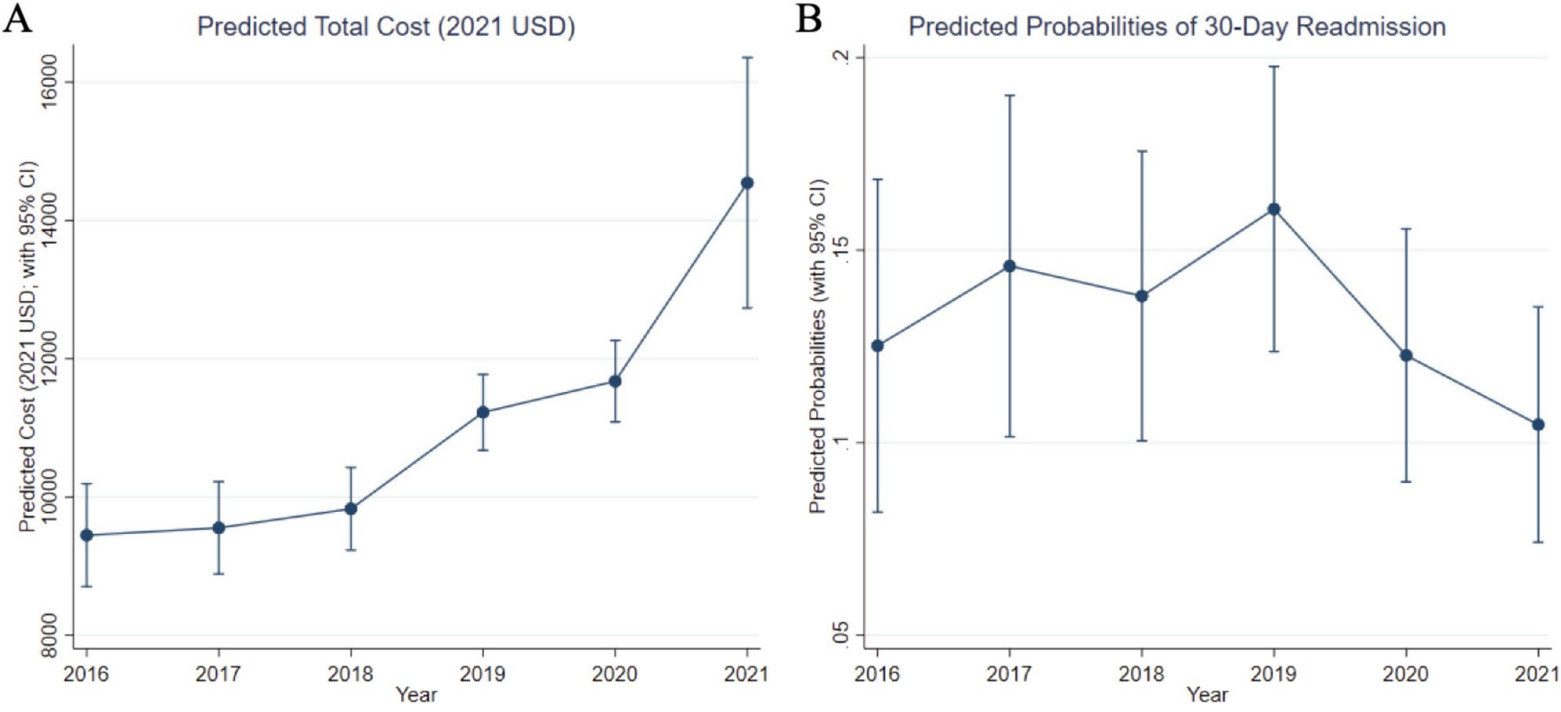
Predicted total admission cost (**A**) and 30-day readmission **(B).** Error bars indicate 95% confidence interval

There were significant differences in the risk-adjusted annual total index cost of care over the study period. The risk-adjusted marginal cost across the years ranged from $9,447 in 2016 to $14,544 in 2021 with a consistent year-over-year increase (Spearman’s correlation test rho = 1, *p*-value = 0.003) (Table 5, Fig. 3a). The risk-adjusted 30-day odds of readmission ranged from 11 to 16% (Table 5, Fig. 3b). Correlational testing demonstrated no significant trend over time for 30-day readmission (Spearman’s correlation test rho = − 0.49, *p*-value = 0.36).

**Table 5:** Marginal estimates of cost and readmissions, by year

Year	Cost*^±^		Readmission**^±^	
	Margins (USD)	95% CI	Margins (%)	95% CI
2016	9,447	8,702, 10,192	0.13	0.08, 0.17
2017	9,553	8,884, 10,222	0.15	0.10, 0.19
2018	9,829	9,232, 10,426	0.14	0.10, 0.18
2019	11,225	10,675, 11,774	0.16	0.12, 0.19
2020	11,675	11,086, 12,264	0.12	0.09, 0.16
2021	14,544	12,734, 16,354	0.11	0.07, 0.14

Models included adjustment for admission year, state, race, sex, insurance class, income quartile, open surgical technique, and Elixhauser comorbidities

*CI* confidence interval, *SD* standard deviation, *USD* United States dollars

^*^Spearman’s correlation test rho = 1, *p*-value = 0.003

^**^Spearman’s correlation test rho = − 0.49, *p*-value = 0.36

** ± **All *p* < 0.001

## Discussion

Using a state-specific geographically representative cohort, we identified significant trends in the utilization and cost of ORC. A significant annual increased proportion of right colectomies occurred in the outpatient setting with a concomitant increase in inflation-adjusted index admission cost over the study period. However, we did not find any differences across the six-year period for 30-day readmission rate.

Our study is the first multi-institutional, multistate study focusing on contemporary trends in the use of ORC, assessing both costs and readmission. Additionally, we present the largest cohort to date of patients undergoing ORC (including open and MIS procedures), allowing us greater power to detect trends. Previous studies have been limited to minimally invasive colectomy type with cohorts in the several tens [9, 20, 21] or hundreds [12–14] of patients.

We found no year-to-year change in the rates of readmission among patients undergoing ORC. However, whereas prospective studies cite readmission percentages around 4–9% (weighted average 5% [14]), our analysis demonstrated twice the rate with an unadjusted median of 12.9%. This may be due, in part, to the fact that we included outpatient open colectomy codes and patients undergoing this procedure have greater readmission rates [22]. However, despite adjustment for surgical approach, our findings maintained a predicted readmission rate ranging from 11 to 16% across all years. Our results may also represent a more heterogenous cohort of patients than previously published single-center studies and may therefore reflect regional variation in readmission. Further, while we did not differentiate between same day and overnight “outpatient” status, published studies have shown similar readmission rates between these two groups of outpatient colectomy patients. [10, 23]

Both domestic and international colorectal groups have reported on the financial implications of short-stay colectomies relative to conventional discharge strategies with significant cost savings demonstrated within multiple reimbursement environments [10, 15, 23]. For instance, a robust analysis performed for same day colectomy (*n* = 143 patients) at a single institution over two years found same day surgery, compared to longer stay, was associated with decreased total and direct cost, but no difference in contribution margin for the hospital [23]. They cited a cost savings of over $3,400 for the total admission cost and average charge savings of over $11,400. Additionally, a recent retrospective analysis looking at same-day discharge (postoperative day 0) for minimally invasive colectomy found that patients discharged on the day of surgery had a significantly lower median cost of index hospitalization compared to those with short-term stay (length of stay ≤ 2 days) [10]. Our findings add to the body of literature for the financial implications of a shift in colectomy care to the outpatient setting by assessing within-year cost changes; we show a significant annual increase in spend per index admission, with costs growing from approximately $8,500 per admission in 2016 to $11,200 in 2021. This reflects differences in costs for outpatient right colectomy patients and not a comparison to costs relative to inpatient colectomy.

Multiple cost drivers within the outpatient setting may be steering this trend. First, outpatient surgical service costs continue to grow at significant rates, in large part due to increasing facility fees [24]. As insurers seek to vertically integrate by buying physician practices, this cost trend may expand into the ambulatory surgical space [25]. Secondly, the increased penetrance of robotic surgery in colorectal surgery certainly could drive inflated admission index costs [26]. Lastly, the need for intensive post-procedural monitoring is capital intensive for hospitals, leading to increasing costs. Ambulatory colectomy protocols have utilized mobile health or telephone remote post-discharge follow up, which require both personnel and technologic infrastructure [1, 27]. Hospitals need to consider the resource overhead prior to the implementation of an ambulatory surgical program, in order to ensure both financial solvency, but most importantly to ensure optimized surgical outcomes given inherent needs for remote patient monitoring.

Several limitations exist within this work and should therefore be discussed. First, given the nature of select state-based coding within SID and SASD, trends enumerated in our study may not be generalizable at the national level. However, we present a geographically diverse set of states, whose similar composition have been used in prior publications to represent national trends [28]. Secondly, given state-based coding, readmissions may have been missed if patients presented to a hospital in a state that was not contained within HCUP. Third, the charge and cost data presented within HCUP does not provide granular detail, so we could not delineate which features of a patient’s admission were driving the increased yearly costs. For instance, we were not able to discern which of the MIS cases were robotic-assisted, due to coding limitations, limiting our cost understanding. We are also unable to know which cases were converted to open. However, these patients are unlikely to have been discharged home early and so if they were included in the MIS group due to misclassification, it would bias our analysis towards the null hypothesis. Lastly, we were unable to control for the number of perioperative resources available to each patient in our analysis, due to limitations of the database. We know that successful programs utilizing outpatient colectomy uniformly have a robust infrastructure of patient support including medical staff to closely follow up with patients, home nursing, and telehealth programs.

In conclusion, this study highlights the growing trend in adoption of outpatient right colectomy across a modern six-year period. We demonstrate that despite this, there was no change in annual readmission rate during this time however there was a significant increase in annual index admission cost. As providers continue to perform safer surgery, patients prefer shorter hospital-stays, hospitals provide greater perioperative home care resources, and policy makers continue to limit cost by shifting care into the outpatient setting, it will be important to continue to monitor these trends, ensuring that outcomes remain optimized. Similarly, our work has implications for future work aimed at elucidating cost drivers in the outpatient setting, as we know that charges for outpatient service fees continue to swell [29]. Nonetheless, it continues to appear as though outpatient right colectomy, in the appropriate patient and setting, remains feasible. Surgeons need to select patients in a rigorously thoughtful manner and policy makers need to continually consider how utilization features impact the cost of outpatient surgery.

## Supplementary Information

Below is the link to the electronic supplementary material.

Supplementary file1 (DOCX 31 KB)
