# Supplementary material for: Temporal trends in outpatient right colectomy: a contemporary multistate study
Source: Surg Endosc. 2025 Jul 14;39(9):6108–16. doi: 10.1007/s00464-025-11888-x (PMC12408695; doi:10.1007/s00464-025-11888-x)
Supplement: Supplementary file 1 — Supplementary file1 (DOCX 31 KB) [file 464_2025_11888_MOESM1_ESM.docx]

Supplementary Data

Table 1: Procedure codes 2

Table 2: All Patient Elixhauser Comorbidities 7

Table 3: Patients Stratified by Length of Stay, Zero Days and One Day 10

Table 1: Procedure codes

| **Feature Type** | **Code** | **Code Type** | **Open/MIS** | **Description** |
| --- | --- | --- | --- | --- |
| Procedure | 0DBF0ZZ | ICD-10 PR | Open | Excision of Right Large Intestine, Open Approach |
| Procedure | 0DBH0ZZ | ICD-10 PR | Open | Excision of Cecum, Open Approach |
| Procedure | 0DBH0ZZ | ICD-10 PR | Open | Excision of Cecum, Open Approach |
| Procedure | 0DBL0ZZ | ICD-10 PR | Open | Excision of Transverse Colon, Open Approach |
| Procedure | 0DBF0ZX | ICD-10 PR | Open | Excision of Right Large Intestine, Open Approach, Diagnostic |
| Procedure | 0DTF0ZZ | ICD-10 PR | Open | Resection of Right Large Intestine, Open Approach |
| Procedure | 0DBK0ZZ | ICD-10 PR | Open | Excision of Ascending Colon, Open Approach |
| Procedure | 0DBL0ZX | ICD-10 PR | Open | Excision of Transverse Colon, Open Approach, Diagnostic |
| Procedure | 0DTK0ZZ | ICD-10 PR | Open | Resection of Ascending Colon, Open Approach |
| Procedure | 0DTL0ZZ | ICD-10 PR | Open | Resection of Transverse Colon, Open Approach |
| Procedure | 0DBK4ZZ | ICD-10 PR | MIS | Excision of Ascending Colon, Perc Endo Approach |
| Procedure | 0DBF4ZX | ICD-10 PR | MIS | Excision of Right Large Intestine, Perc Endo Approach, Diagn |
| Procedure | 0DBH4ZX | ICD-10 PR | MIS | Excision of Cecum, Percutaneous Endoscopic Approach, Diagn |
| Procedure | 0DBL4ZX | ICD-10 PR | MIS | Excision of Transverse Colon, Perc Endo Approach, Diagn |
| Procedure | 0DTH4ZZ | ICD-10 PR | MIS | Resection of Cecum, Percutaneous Endoscopic Approach |
| Procedure | 0DTK4ZZ | ICD-10 PR | MIS | Resection of Ascending Colon, Perc Endo Approach |
| Procedure | 0DTF4ZZ | ICD-10 PR | MIS | Resection of Right Large Intestine, Perc Endo Approach |
| Procedure | 0DTL4ZZ | ICD-10 PR | MIS | Resection of Transverse Colon, Perc Endo Approach |
| Procedure | 0DTF7ZZ | ICD-10 PR | MIS | Resection of Right Large Intestine, Via Opening |
| Procedure | 0DBF4ZZ | ICD-10 PR | MIS | Excision of Right Large Intestine, Perc Endo Approach |
| Procedure | 0DBF7ZZ | ICD-10 PR | MIS | Excision of Right Large Intestine, Via Opening |
| Procedure | 0DBH4ZZ | ICD-10 PR | MIS | Excision of Cecum, Percutaneous Endoscopic Approach |
| Procedure | 0DBH7ZZ | ICD-10 PR | MIS | Excision of Cecum, Via Natural or Artificial Opening |
| Procedure | 0DBK7ZZ | ICD-10 PR | MIS | Excision of Ascending Colon, Via Opening |
| Procedure | 0DBL4ZZ | ICD-10 PR | MIS | Excision of Transverse Colon, Perc Endo Approach |
| Procedure | 0DBL7ZZ | ICD-10 PR | MIS | Excision of Transverse Colon, Via Opening |
| Procedure | 0DTH7ZZ | ICD-10 PR | MIS | Resection of Cecum, Via Natural or Artificial Opening |
| Procedure | 0DTK7ZZ | ICD-10 PR | MIS | Resection of Ascending Colon, Via Opening |
| Procedure | 0DTL7ZZ | ICD-10 PR | MIS | Resection of Transverse Colon, Via Opening |
| Procedure | 0DTF8ZZ | ICD-10 PR | MIS | Resection of Right Large Intestine, Endo |
| Procedure | 44140 | CPT | Open | Colectomy, partial; with anastomosis |
| Procedure | 44141 | CPT | Open | Colectomy, partial; with skin level cecostomy or colostomy |
| Procedure | 44160 | CPT | Open | Colectomy, partial, with removal of terminal ileum with ileocolostomy |
| Procedure | 44204 | CPT | MIS | Laparoscopy, surgical; colectomy, partial, with anastomosis |
| Procedure | 44205 | CPT | MIS | Laparoscopy, surgical; colectomy, partial, with removal of terminal ileum with ileocolostomy |
| CPT=Common Procedural Terminology, PR=procedure code | | | | |

Table 2: All Patient Elixhauser Comorbidities

|  | **All Years** | **2016** | **2017** | **2018** | **2019** | **2020** | **2021** |
| --- | --- | --- | --- | --- | --- | --- | --- |
| AIDS, N (%) | * | 0 (0) | 0 (0) | * | 0 (0) | * | * |
| Alcohol, N (%) | * | * | * | * | 0 (0) | 0 (0) | * |
| Iron deficiency anemia, N (%) | 112 (5.8) | * | * | 20 (6.2) | 16 (4.4) | 28 (7.6) | 28 (7.3) |
| Arthropathy, N (%) | 40 (2.1) | * | * | * | * | * | * |
| Blood loss anemia | * | * | 0 (0) | * | * | * | * |
| Lymphoma, N (%) | * | 0 (0) | * | * | 0 (0) | 0 (0) | * |
| Leukemia, N (%) | * | * | 0 (0) | * | 0 (0) | 0 (0) | * |
| Cancer, solid with metastases, N (%) | 67 (3.5) | * | * | * | * | 18 (4.9) | 20 (5.2) |
| Cancer, solid without metastases, N (%) | 58 (3) | * | * | * | * | 12 (3.2) | 13 (3.4) |
| Cancer, solid in-situ, N (%) | * | * | 0 (0) | 0 (0) | 0 (0) | 0 (0) | 0 (0) |
| Cerebrovascular disease, N (%) | 12 (0.6) | * | * | * | * | * | * |
| Heart Failure, N (%) | 30 (1.6) | * | * | * | * | * | * |
| Coagulopathy, N (%) | * | 0 (0) | * | * | 0 (0) | * | * |
| Dementia, N (%) | * | * | 0 (0) | 0 (0) | * | * | 0 (0) |
| Depression, N (%) | 165 (8.5) | 21 (8.6) | 20 (8.1) | 28 (8.7) | 33 (9) | 32 (8.7) | 31 (8.1) |
| Diabetes, uncomplicated, N (%) | 238 (12.2) | 27 (10.8) | 30 (11.9) | 40 (12.3) | 47 (12.7) | 44 (11.8) | 50 (13.1) |
| Diabetes, complicated, N (%) | 93 (4.8) | * | * | 18 (5.6) | 19 (5.2) | 15 (4.1) | 22 (5.8) |
| Hypertension, uncomplicated, N (%) | 778 (39.8) | 99 (39.4) | 95 (37.7) | 138 (42.5) | 142 (38.3) | 147 (39.5) | 157 (41) |
| Hypertension, complicated, N (%) | 101 (5.2) | * | * | * | 29 (7.9) | 17 (4.6) | 23 (6.0) |
| Liver disease, mild, N (%) | 43 (2.2) | * | * | * | * | * | 14 (3.7) |
| Liver disease, severe, N (%) | * | 0 (0) | 0 (0) | 0 (0) | 0 (0) | 0 (0) | * |
| Chronic lung disease, N (%) | 234 (12.1) | 25 (10.2) | 37 (115) | 42 (13.1) | 49 (13.3) | 38 (10.3) | 43 (11.3) |
| Movement disorder, N (%) | 14 (0.72) | 0 (0) | * | * | * | * | * |
| Neurologic disease, other, N (%) | * | 0 (0) | * | * | * | 0 (0) | * |
| Seizure disorder, N (%) | 14 (0.72) | * | 0 (0) | * | * | * | * |
| Obesity, N (%) | 303 (15.7) | 31 (12.7) | 34 (13.8) | 57 (17.8) | 56 (15.2) | 55 (14.9) | 70 (18.3) |
| Paralysis, N (%) | * | 0 (0) | * | * | * | * | * |
| Peripheral vascular disease, N (%) | 50 (2.6) | * | * | * | * | * | 15 (3.9) |
| Psychoses, N (%) | 14 (0.7) | 0 (0) | * | * | * | * | * |
| Pulmonary circulatory disorder, N (%) | * | * | * | * | 0 (0) | 0 (0) | * |
| Renal failure, moderate, N (%) | 49 (2.5) | * | * | * | * | * | 13 (3.4) |
| Renal failure, severe, N (%) | * | 0 (0) | 0 (0) | * | * | * | 0 (0) |
| Hypothyroid, N (%) | 174 (9) | 20 (8.2) | 25 (10.1) | 31 (9.7) | 35 (9.5) | 31 (8.4) | 32 (8.4) |
| Thyroid disorder, other, N (%) | 15 (0.8) | * | * | * | * | * | * |
| Peptic ulcer disease, N (%) | * | 0 (0) | 0 (0) | * | 0 (0) | 0 (0) | 0 (0) |
| Valvular disorder, N (%) | 38 (2) | * | * | * | * | * | * |
| Weight loss | * | * | * | 0 (0) | * | * | 0 (0) |
| All p>0.05  *Censored due to HCUP data use agreement  AIDS=acquired immune deficiency syndrome | | | | | | | |

Table 3: Patients Stratified by Length of Stay, Zero Days and One Day

|  | All Years | 2016 | 2017 | 2018 | 2019 | 2020 | 2021 | p-value* |
| --- | --- | --- | --- | --- | --- | --- | --- | --- |
| Length of Stay 0 Days, N (%) | 523 (27.1%) | 72 (29.4%) | 65 (26.3%) | 91 (28.4%) | 118 (32.1%) | 87 (23.5%) | 90 (23.6%) | 0.06 |
| Length of Stay 1 Day, N (%) | 1,410 (72.9%) | 173 (70.6%) | 182 (73.7%) | 230 (71.7%) | 250 (67.9%) | 283 (76.5%) | 292 (76.4%) |  |
| Total | 1,933 | 245 | 247 | 321 | 368 | 370 | 382 |  |
| *Chi-squared test | | | | | | | | |
